# Drivers of topoisomerase II poisoning mimic and complement cytotoxicity in AML cells

**DOI:** 10.18632/oncotarget.27112

**Published:** 2019-09-03

**Authors:** Piyush More, Ute Goedtel-Armbrust, Viral Shah, Marianne Mathaes, Thomas Kindler, Miguel A. Andrade-Navarro, Leszek Wojnowski

**Affiliations:** ^1^ Department of Pharmacology, University Medical Center, Johannes Gutenberg University Mainz, Mainz, Germany; ^2^ Department of Hematology, Medical Oncology and Pneumology, University Medical Center, Johannes Gutenberg University Mainz, Mainz, Germany; ^3^ University Cancer Center of Mainz, Mainz, Germany; ^4^ Computational Biology and Data Mining, Faculty of Biology, Johannes Gutenberg University Mainz, Mainz, Germany

**Keywords:** topoisomerase II poisons, DNA damage, gene expression, combination therapy, cancer essentiality

## Abstract

Recently approved cancer drugs remain out-of-reach to most patients due to prohibitive costs and only few produce clinically meaningful benefits. An untapped alternative is to enhance the efficacy and safety of existing cancer drugs. We hypothesized that the response to topoisomerase II poisons, a very successful group of cancer drugs, can be improved by considering treatment-associated transcript levels. To this end, we analyzed transcriptomes from Acute Myeloid Leukemia (AML) cell lines treated with the topoisomerase II poison etoposide. Using complementary criteria of co-regulation within networks and of essentiality for cell survival, we identified and functionally confirmed 11 druggable drivers of etoposide cytotoxicity. Drivers with pre-treatment expression predicting etoposide response (e.g., PARP9) generally synergized with etoposide. Drivers repressed by etoposide (e.g., PLK1) displayed standalone cytotoxicity. Drivers, whose modulation evoked etoposide-like gene expression changes (e.g., mTOR), were cytotoxic both alone and in combination with etoposide. In summary, both pre-treatment gene expression and treatment-driven changes contribute to the cell killing effect of etoposide. Such targets can be tweaked to enhance the efficacy of etoposide. This strategy can be used to identify combination partners or even replacements for other classical anticancer drugs, especially those interfering with DNA integrity and transcription.

## INTRODUCTION

Topoisomerase II (TOP2) poisons belong to the most efficient class of anti-cancer drugs. They prevent re-ligation of otherwise transient DNA single and double strand breaks generated by TOP2, ultimately triggering apoptosis [1, 2]. Unfortunately, due to the involvement of TOP2 in such fundamental cellular processes as DNA replication and transcription, its poisoning affects both cancerous and normal cells. Thus, in addition to the transient bone marrow toxicity, TOP2 poisons cause irreversible side-effects such as secondary leukemia [3] and cardiotoxicity [4]. They are, therefore, gradually being supplemented by drugs targeting molecules and processes more specific to cancer cells.

Taking AML as an example, midostaurin and enasidenib can be nowadays added to standard chemotherapeutic regimens [5, 6] in patients carrying specific mutations in the protein targets of these drugs, FLT3 and IDH2, respectively. It is expected that, eventually, each cancer patient’s molecular tumor profile will be matched to a tailored treatment regimen. However, reaching the point where we can do this will take many years, as drugs targeting such individual targets will have to be developed, tested, and approved for even smaller patient cohorts. Furthermore, new cancer drugs remain out-of-reach to most patients due to prohibitive costs and they confer rather modest clinical benefits [7].

Here we explore the alternative and largely untested approach of fine-tuning established cancer therapies by combining them with already approved or experimental drugs targeting their cytotoxicity drivers. To this end, we analyzed gene expression profiles preceding and following the exposure to TOP2 poison etoposide. We reasoned that: (i) these profiles co-determine cell-killing effects of TOP2 poisons; and (ii) drugs targeting some of the involved genes’ protein products would be already available for testing as combination partners. We chose the TOP2 poison etoposide as a case scenario since apoptosis resulting from etoposide-driven DNA damage is accompanied by considerable gene expression changes of unexplored consequences [8, 9]. Furthermore, etoposide acts exclusively via TOP2, in contrast to anthracyclines, which additionally intercalate with DNA and target cellular mitochondria [10].

We chose AML as a cancer model, since AML is frequently treated with etoposide, especially relapsed cases [11]. We employed transcription analysis, since this is currently the most sensitive technique interrogating the expression of all genes, while broadly correlating with expression levels of their protein products. We assessed etoposide-driven gene expression changes by comparing pre-and post-treatment cell transcriptomes. We also considered the impact of prior-to-treatment gene expression levels on the response to etoposide across AML cell lines. Here, we reasoned that, in addition to expression *changes*, the response to etoposide is likely to be affected by pre-existing levels of proteins modulating its effects.

Tumor growth and metastasis are driven only by a fraction of the accompanying molecular changes. We assumed a similar relationship for etoposide response and gene expression levels. We intended to enrich for drivers as opposed by bystanders of etoposide cytotoxicity using two parallel approaches. Firstly, we focused on genes co-regulated within networks correlating with etoposide cytotoxicity. Here, we reasoned that genes involved in such networks are more likely to be involved in etoposide response compared to genes taken individually [12, 13]. Secondly, we focused on individual, but essential genes, i.e., on those reducing the survival of each of the AML cell lines investigated when knocked down using shRNA [14]. Among drivers thus identified, we differentiated between modulators, mediators, and emulators of etoposide response. Etoposide modulators are genes, whose expression correlates with etoposide cytotoxicity, but remains unchanged upon treatment. Etoposide mediators are genes that convey cytotoxicity via etoposide-driven changes in their expression levels. Etoposide emulators are upstream gene modulations and other drugs that evoke gene expression profiles resembling those evoked by etoposide.

## RESULTS

### Overview of the pipeline

**Figure 1:**
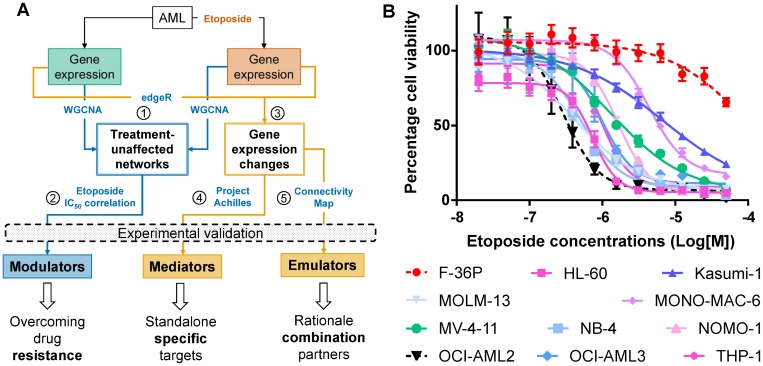
Identifying drivers of differential etoposide sensitivity in AML cell lines. (**A**) Pipeline integrating experimental data with publicly available resources to identify drivers of etoposide-mediated cytotoxicity. Numbers in circles identify major procedural steps (See Results section for details). (**B**) Concentration-dependent effect of etoposide on survival of AML cell lines after 24 hours. The most (OCI-AML2) and the least (F-36P) sensitive cell lines are depicted in black and red dotted lines respectively.

To identify drugs that could supplement or replace etoposide, we determined, analyzed, and functionally verified gene expression profiles prior and after etoposide treatment (Figure 1A). Two parallel approaches were followed. First, we identified networks of co-regulated genes (step 1 in Figure 1A). Genes derived from these networks, whose co-regulation was unaffected by etoposide and whose expression correlated with etoposide IC_50_, were defined as potential modulators of etoposide cytotoxicity (step 2). Second, among the etoposide-evoked individual gene expression changes (step 3), the essential genes were identified by applying the PAch-derived cancer cell essentiality filter (step 4). Putative etoposide emulators, i.e., gene modulations and drugs that cause gene expression changes either similar or contrary to those evoked by etoposide, were identified using CMap (step 5). Putative modulators, effectors, and emulators thus identified were further analyzed based on their: (i) biological function; (ii) relevance to a majority of AML cell lines; and (iii) inhibitor availability, and subjected to functional validation.

### AML cell lines differ in etoposide sensitivity

We applied the WST8 cell viability assay to 11 AML cell lines following 24 hours of exposure to 0.02–50 μM etoposide. The cell lines exhibited differential sensitivity to etoposide (Figure 1B, Supplementary Table 1), with IC_50_ concentrations ranging from 0.3 μM (OCI-AML2) to 99 μM (F-36P). Since WST8 assay rely on metabolic activity of cells, we validated the IC_50_ concentrations by measuring apoptosis with Annexin V-FITC staining. The percentages of dead cells at WST8-derived IC_50_ values differed, on average, by 16% (1.4–30.6%) from percentages of apoptotic cells in the same cell lines treated identically but interrogated using Annexin V-FITC (Supplementary Figure 1).

### Modulators synergize with etoposide

**Figure 2:**
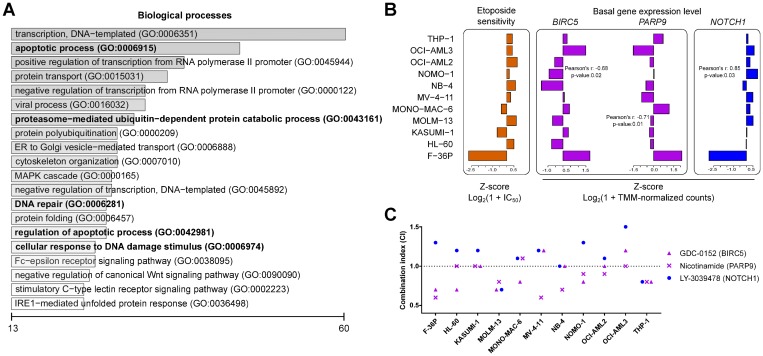
Impeding modulators synergize with etoposide. (**A**) Top 20 biological processes for the co-expressed genes from the consensus network negatively correlating with etoposide sensitivity. The scale represents number of genes enriched for individual biological processes. Processes previously linked to etoposide are shown in bold type. (**B**) Pearson correlations between the pre-treatment basal gene expression level of the impeding modulators *BIRC5* and *PARP9* and of the assisting modulator *NOTCH1* with etoposide sensitivity across AML cell lines. (**C**) Combination index (CI; see Methods for details) for the cytotoxicity following treatment with IC_25_ concentrations of etoposide with inhibitors targeting the impeding modulators BIRC5 and PARP9 and the assisting modulator NOTCH1. CI < 1: synergism, CI = 1: additivity, and CI > 1: antagonism.

The AML cell lines were then treated for 24 hours with cell line-specific IC_50_ concentrations of etoposide to obtain similar cytotoxicity levels. We discarded RNA-Seq data from etoposide-treated OCI-AML2 cells, because it failed in the quality control of raw RNA sequences. Using WGCNA, we identified genes co-regulated in all 11 untreated AML cell lines, as well as in the remaining 10 etoposide-treated cell lines. By comparing pre- and post-treatment networks, we first identified and analyzed the genes with co-regulation unaffected by the treatment (Supplementary Figure 2). The 24 treatment-unaffected clusters comprised 5711 genes. The genes with expression levels correlating with etoposide response were involved in processes such as apoptosis, proteasomal catabolism, response to DNA damage, and DNA repair (Figure 2A and Supplementary Table 3). The 71 genes correlating positively with etoposide IC_50_ concentrations were considered putative *assisting* modulators; the 909 negatively correlating ones as putative impeding modulators (p < 0.05, Pearson’s r > |0.5|, Supplementary Table 3). Among them, we identified the previously reported modulators *SLFN11* [15, 16] and *SMARCA4* [17] whose expression correlated with etoposide sensitivity (Supplementary Table 3).

The putative impeding modulators *BIRC5* and *PARP9* (Figure 2B) were selected for experimental validation using chemical inhibitors against their protein products because of their involvement in apoptosis regulation and in double strand break repair, respectively. *NOTCH1* (Figure 2B) was selected for experimental validation to confirm its putative etoposide-assisting activity. AML cell lines were treated for 24 hours with 3 concentrations (0.001 μM, 0.1 μM, and 10 μM) of chemical inhibitors alone, as well as in combination with cell line-specific IC_25_ concentrations of etoposide. The BIRC5 inhibitor GDC-0152 and the PARP inhibitor nicotinamide exhibited effects synergistic or additive to etoposide in 9 and 10 cell lines, respectively (Figure 2C and Table 1). The NOTCH1 inhibitor LY-3039478 antagonized with etoposide in 8 out of 11 AML cell lines (Figure 2C, Table 1, and Supplementary Table 4). Stand-alone cytotoxicity was observed in OCI-AML3 cells following BIRC5 inhibition and in two cell lines following NOTCH1 inhibition (Table 1 and Supplementary Table 5). In summary, all putative modulators investigated were confirmed by chemical inhibitors.

**Table 1:** Drivers of etoposide cytotoxicity identified in this study

Drivers type	Targets (inhibitors)	Standalone cytotoxicity (no. of cell lines)	Synergy/additivity with etoposide (no. of cell lines)
**Modulators**	NOTCH1 (LY-3039478)	2	2
	BIRC5 (GDC-0152)	1	**9**
	PARP9 (Nicotinamide)	0	**10**
**Mediators**	BCL2A1 (Sabutoclax)	**11**	1
	PRKCH (Sotrastaurin)	**7**	3
	PLK1 (Volasertib)	**11**	1
	IGF1R (GSK-1838705A)	**9**	2
**Emulators**	MYC (TWS-119)	**10**	2
	mTORi (Rapamycin)	**7**	**6**
	HDACi (Vorinostat)	**9**	**9**
	ROCK1 (Rockout)	3	**7**

The drivers exhibiting stand-alone cytotoxicity in at least 6 AML cell lines are highlighted in light grey, drivers synergizing with etoposide in at least 6 AML cell lines in dark grey.

### Mediators exhibit standalone cytotoxicity

We next analyzed co-regulated genes with expression levels correlating with the etoposide IC_50_ concentrations, but transcriptionally *altered* by etoposide treatment. The co-regulated genes found only in untreated cells, e.g., *BRD4*, *MATL1*, and *MYC,* regulate, among others, cell proliferation, transcription, and apoptosis (Supplementary Table 6). The genes co-regulated only in networks newly formed after etoposide treatment, e.g., *SIRT1,* regulate, among others, transcription, response to DNA damage, and DNA repair (Supplementary Table 7). *BRD4* and *MYC* were transcriptionally repressed, while *MALT1* and *SIRT1* were transcriptionally induced by etoposide in the less responsive AML cell lines (Supplementary Figure 3). However, all of them, except *MYC*, were essential in only 4 AML cell lines, based on their DEMETER scores.

**Figure 3:**
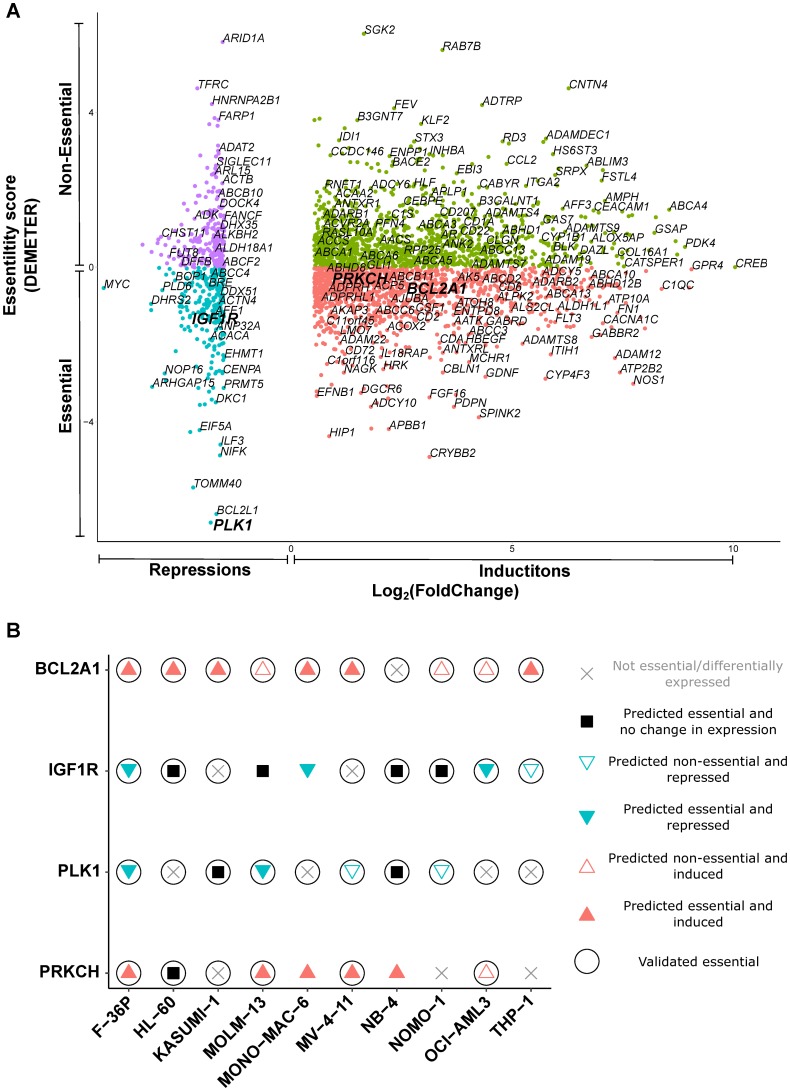
Essential mediators exert cytotoxicity in AML cell lines. (**A**) Scatterplot of etoposide-evoked differentially expressed genes in F-36P cell line, arranged according to essentiality for survival. DEMETER score < 0 signifies essentiality. The genes essential for tumor cell survival and differentially expressed after etoposide treatment were considered as putative essential mediators. The mediators shortlisted for experimental validation (*BCL2A1*, *IGF1R*, *PLK1*, and *PRKCH)* are depicted in larger font. Other gene names are random examples taken from the entire gene set. (**B**) Experimental validation of putative essential mediators shortlisted in (A). Cell viability was assessed by WST-8 assay after treatment with inhibitors targeting protein products of shortlisted drivers. Filled symbols represent predicted essentiality for survival in individual AML cell lines. Circles around the symbols represent experimentally confirmed cytotoxicity.

Therefore, we next analyzed and functionally verified etoposide-driven gene expression changes at the level of individual genes. Gene inductions accounted for 81% of etoposide treatment-driven transcriptional changes (Supplementary Table 8). Essentiality analysis suggested that, on average, about 33% of etoposide-driven changes could have reduced AML cell survival (Supplementary Table 9). An example of gene expression changes grouped according to essentiality in F-36P cell line is shown in Figure 3A. We selected *IGF1R* for experimental validation, since it was essential for 7 AML cell lines and repressed in 4 AML cell lines after etoposide treatment (Figure 3B and Supplementary Table 10). Likewise, *PLK1*, was essential as well as repressed in 4 AML cell lines (Figure 3B and Supplementary Table 10). We pursued *PLK1* because it exhibited highest essentiality for the least etoposide-sensitive F-36P cell line (Figure 3A and Supplementary Table 10). *BCL2A1* and *PRKCH* were selected because of their predicted essentiality for 6 AML cell lines each, and because they were induced by etoposide in 9 and 6 AML cell lines, respectively (Figure 3B and Supplementary Table 10).

We treated all AML cell lines with the inhibitors of the protein products of these genes alone, as well as in combination with IC_25_ concentrations of etoposide. The inhibitors targeting the protein products of *BCL2A1* and *PLK1* exerted standalone cytotoxicity in all AML cell lines, while the IGF1R inhibitor and the PKC inhibitor exhibited cytotoxicity in 9 and 7 AML cell lines, respectively (Figure 3B, Table 1, and Supplementary Figure 4). Inhibition of BCL2A1 and PLK1 synergized with etoposide in MOLM-13 and NB-4 cell lines, respectively. Inhibition of PRKCH and IGF1R exhibited synergy with etoposide in 2 AML cell lines each (Table 1 and Supplementary Table 4). We additionally investigated in HL-60 cells the cytotoxic effects of the essential mediators BCL2A1 and IGF1R using shRNA-mediated knockdown. Knockdown of the mediator IGF1R was cytotoxic to HL-60 cells. (Supplementary Figure 5). In summary, all putative etoposide mediators investigated were confirmed to be cytotoxic in most AML cell lines.

### Emulators are cytotoxic and synergize with etoposide

**Figure 4:**
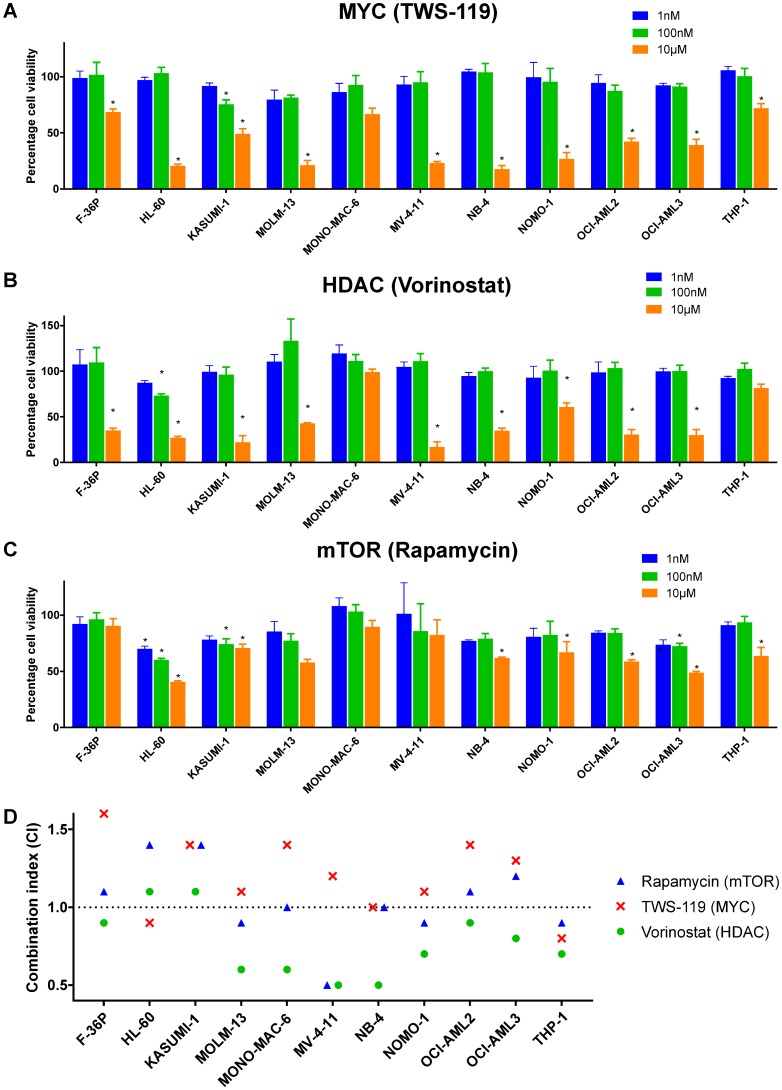
Etoposide-like emulators are cytotoxic and synergize with etoposide. Concentration-dependent cytotoxicity after inhibiting etoposide-like emulators (**A**) MYC with TWS-119, (**B**) HDAC with vorinostat, and (**C**) mTOR with rapamycin. (**D**) Combination index (CI; see Methods for details) of etoposide treatment with protein inhibitors targeting MYC, HDAC, and mTOR. CI < 1: synergism, CI = 1: additivity, and CI > 1: antagonism. Two-way ANOVA with Benjamini and Hochberg FDR correction was performed to identify statistically significant cytotoxicity in comparison to vehicle treated cells (indicated by asterisks, ^*^Adj. *P* < 0.05). Data are represented as mean ± SD.

Using the CMap resource, we identified gene modulations and drugs that cause gene expression changes either similar or contrary to those evoked by etoposide. There were 32 gene knockdowns and 76 drugs whose application led to etoposide-like gene expression changes. They were referred to as putative etoposide-*like* emulators. The majority of the drugs belonged to the classes mTOR inhibition, topoisomerase inhibition, and HDAC inhibition. We also identified 12 drugs evoking opposite gene expression changes, referred to as putative etoposide-*contrary* emulators (Supplementary Table 11). We measured cell viability in AML cell lines treated with inhibitors targeting the protein products of selected putative etoposide-like emulators individually, as well as in combination with etoposide (IC_25_ concentrations) for 24 hours. Targeting of the etoposide-like emulator *MYC* with TWS-119 led to cytotoxicity in all AML cell lines except MONO-MAC-6 (Figure 4A and Table 1). Similarly, inhibition of etoposide-like emulators HDAC with vorinostat and of mTOR with rapamycin evoked cell death in in 9 and 6 AML cell lines, respectively (Figure 4B and 4C, and Table 1). Interestingly, vorinostat and rapamycin also exhibited synergy or additivity with etoposide in 9 and 6 AML cell lines, respectively (Figure 4D, Table 1, and Supplementary Table 4).

The etoposide-contrary emulator *ROCK1* also synergized or exhibited additivity with etoposide in 7 out of 11 AML cell lines, when inhibited with rockout. Inhibition of ROCK1 was cytotoxic in only 3 AML cell lines (Table 1 and Supplementary Table 5). The target specificity of rockout was confirmed by demonstrating cytotoxicity in HL-60 cells upon shRNA-mediated knockdown of *ROCK1* (Supplementary Figure 5).

### Driver-etoposide combinations enhance cytotoxicity without increasing DNA damage

**Figure 5:**
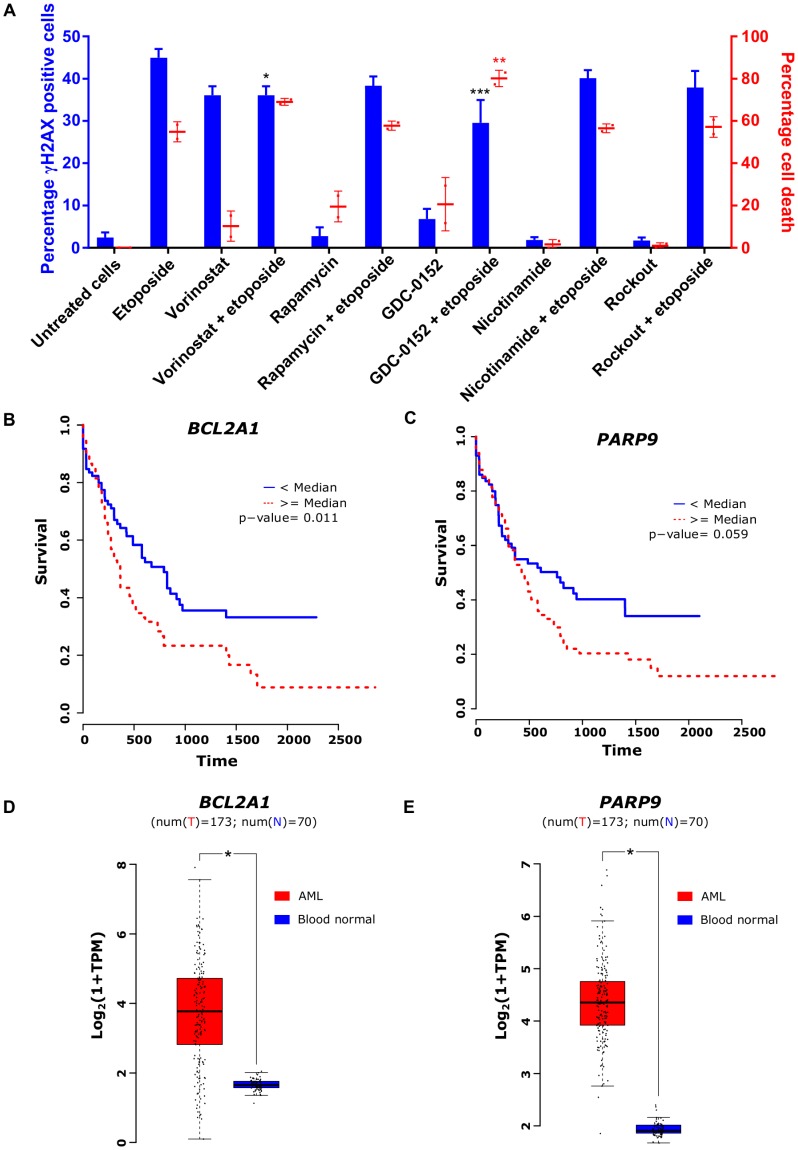
Safety and clinical relevance of transcriptional drivers of etoposide. (**A**) Fraction of phospho-H2A.X-positive cells counted using flow cytometry (black bars) and percentages of cell death after treatment with different inhibitors alone as well as in combination with IC_25_ concentration of etoposide in HL-60 cell line (gray bars). (**B**) and (**C**) Kaplan-Meier plots representing survival of AML patients with high and low expression of *BCL2A1* and *PARP9*, respectively. (**D**) and (**E**) Basal expression of *BCL2A1* and *PARP9*, respectively, in AML and normal blood cells. One-way ANOVA with Dunnett’s multiple comparisons test was performed to identify significant γH2AX formation and cell death induction in comparison to etoposide alone (indicated by asterisks, ^*^Adj. *P* < 0.05, ^***^Adj. *P* < 0.0005). Data are represented as mean ± SD.

To assess the safety of the experimentally validated combinations of etoposide with other drugs, we examined their effect on DNA damage in the HL-60 cell line. We measured the number of FITC-conjugated anti-phospho H2A.X-labelled HL-60 cells by flow cytometry before and after the treatment with etoposide alone or in combination with other drugs for 24 hours. Etoposide caused, as an effect of TOP2-poisoning, DNA damage in 45% of cells at IC_25_ concentration. None of the investigated etoposide-combinations elevated the amount of DNA damage in comparison to etoposide alone (Figure 5A). The BIR inhibitor GDC-0152 even reduced the amount of DNA damage in comparison to etoposide alone.

### Drivers of etoposide cytotoxicity form unfavorable prognostic markers in AML patients

To assess the clinical relevance of identified drivers, we inspected gene expression and clinical data of 173 AML patients from TCGA and compared with gene expression in 30 normal blood samples from GTEx. The analysis revealed an association between high expression of *BCL2A1* and *PARP9* with poor survival in AML patients (Figure 5B and 5C). Furthermore, these genes were highly expressed in AML patients compared to healthy individuals (Figure 5D and 5E). Additionally, the Human Protein Atlas resource revealed high expression of *BIRC5* or *PLK1* to be associated with poor survival in renal, liver, and lung cancer patients and high expression of *ROCK1* to be a marker of unfavorable prognosis in pancreatic cancer [18].

## DISCUSSION

In this work, we demonstrate that etoposide kills cancer cells depending on expression levels of driver genes, some of which it modulates. Since WST8 data correlated well with Annexin V, we speculate that cell loss predominates over growth inhibition. This effect is distinct from the etoposide concentration-driven increase in DNA double stranded breaks [19]. Targeting these drivers genetically or pharmacologically mimics or augments the response to etoposide, indicating a potential for clinical exploration. The pipeline used to discover drivers of etoposide cytotoxicity is applicable to other TOP2 inhibitors and to cytotoxic drugs in general.

### DNA double-strand-breaks independent cytotoxicity of etoposide

Since the response of cancer cells to TOP2 poisons is variable, attempts have been made to explain it by considering pre-treatment gene expression levels [15, 20–23]. SLFN11 [15, 16] and SMARCA4 [17], re-discovered in our study, have been identified as modulators of TOP2 poisons, including etoposide, but failed to make clinical impact. Treatment-driven gene expression changes have been likewise reported [24, 25], but not explored for optimizing response to TOP2 poisons. We considered both pre-treatment gene expression levels *and* drug-evoked changes, as a surrogate of pre- and post-treatment protein expression levels.

Post-treatment transcriptomes were particularly important, since they were essential for the discovery of 8 out of 11 functionally confirmed drivers (i.e., of all mediators and emulators depicted in Table 1). Only gene repressions can be expected to arise directly from DNA damage within regulatory or coding gene sequences. Furthermore, the number of etoposide-driven DNA double strand breaks, assessed by H2A.X, is typically 1–2 orders of magnitude lower than the number of gene expression changes [26]. Hence, most of the observed etoposide-evoked gene expression changes were likely secondary, which is also consistent with the predominance of gene inductions over reductions. Unsurprisingly, these secondary changes partly reflected the activation of DNA damage response.

The filtering strategies used, WGCNA and essentiality analysis, were originally meant to maximize the specificity of driver detection. Genes co-regulated across specimens are more likely to play important roles in, for example, drug response, than individual genes [12, 13]. The same applies to the genes essential for cancer cell survival [14]. This strategy was successful, since all 11 potential drivers identified were functionally confirmed according to pre-set criteria. In addition, combining these two strategies may have improved the sensitivity of our approach. For example, *MYC* missed detection in WGCNA analysis, but was identified as a potential driver in mediator and emulator screens.

We based our classification of etoposide drivers primarily on gene expression, with modulators remaining unchanged, mediators undergoing changes, and emulators mimicking etoposide-like expression profiles. Interestingly, this classification broadly correlates with the functional validation. Thus, mediators displayed standalone cytotoxicity but little synergy with etoposide, modulators behaved inversely, whereas emulators exhibited a mix of both (Table 1). The absence of synergy of etoposide with mediators likely reflects the convergence of etoposide (via expression change) and a mediator’s chemical inhibitor on one and the same target. Conversely, modulators may synergize with etoposide precisely because they remain unaffected by etoposide. Emulators may be cytotoxic alone and synergize with etoposide due to the complex expression changes they evoke.

### Potential application to AML and other cancers

Etoposide effects can clearly be optimized by targeting drivers of its toxicity, but how relevant is this strategy to AML management? AML relapse occurs in around 40% of patients treated with first-line chemotherapy, typically comprising cytarabine and daunorubicin [27]. Although there is no standard treatment, relapsed cases are often treated with mitoxantrone, etoposide, and cytarabine (MEC) combinations. However, many patients do not tolerate the associated increased side-effects [27, 28]. Hence, there is a need to improve efficacy and reduce the toxicity of these treatment regimens. Similar needs exist for other etoposide applications, such as testicular, prostate, and small cell lung cancer.

Interestingly, some of the drivers described in this work have been or are currently undergoing testing. This provides an additional validation of our approach. Supplementing etoposide with the inhibitor of its emulator mTOR with rapamycin has already been shown to reduce the survival of cancer cells in a mouse model of AML [29]. A phase II trial for managing high-risk AML patients with rapamycin in combination with MEC regimen is ongoing (NCT02583893). The etoposide-synergy with HDAC inhibition is currently undergoing testing for Acute Lymphoblastic Leukemia (NCT02553460). Due to the interaction of TOP2 with HDAC1 and 2, etoposide-evoked DNA double strand breaks could affect the chromatin architecture [30]. Interestingly, we observed etoposide-evoked induction in *SIRT1*. It is evident that SIRT1 induction synergizes with HDAC inhibition [31]. This is in agreement with the observed etoposide-evoked induction of *SIRT1* and its observed synergy with vorinostat.

Improved clinical outcomes in AML patients have been already reported for the PLK1 inhibitor volasertib [32] and a Phase III trial is ongoing (NCT01721876). Strikingly, PLK1 inhibition with volasertib was cytotoxic in all 11 AML cell lines. This suggests that cytotoxicity drivers can be efficient beyond the cohort subset in which they were detected. Inhibition of IGF1R (Insulin-like growth factor receptor 1) has been found to be efficacious together with etoposide and cisplatin in small-cell lung cancer [33] and further clinical trials are undergoing with other drugs and cancer types.

The use of DNA damaging drugs, including etoposide, is associated with increased risk of secondary leukemia because of chromosomal aberrations [34, 35]. Hence, it is crucial to formulate combination partners that do not increase the risk even further. Our primary investigation using DNA double strand breaks marker γH2A.X revealed that none of the combinational partners elevated the DNA damage compared to etoposide alone. We speculate that etoposide in combination with its cytotoxicity drivers would exert less side effects because of its dose reduction. However, this needs in-depth investigation using AML mouse models.

### Limitations

The approach used in this study has certain limitations and caveats, beginning with the assumption of gene expression reflecting protein expression. While this assumption is generally true, the expression and activity levels of some proteins are regulated without changes in the RNA expression level. Nevertheless, all potential drivers selected for validation displayed standalone toxicity or modified that of etoposide. Altogether, using transcriptome data is sufficiently sensitive and specific to detect and confirm cytotoxicity drivers worth further exploration in animal models and in the clinic.

Furthermore, it seems that some genes identified as drivers serve as markers of additional, undetected drivers. For example, inhibiting the etoposide modulator BCL2A1 with sabutoclax caused cytotoxicity in all AML cells. In contrast, a shRNA-mediated knockdown of BCL2A1 in HL-60 cells had no effect. Additional members of the Bcl2 family may have contributed to the effect of sabutoclax, a pan-Bcl2 inhibitor. shRNA-mediated knockdowns did confirm the specific involvements of *IGF1R* and *ROCK1*. Wherever possible, putative drivers should undergo verification both by genetic *and* pharmacological means.

### Perspective

Our results suggest a major and yet untapped contribution of etoposide-evoked gene expression changes to this drug’s anti-cancer effects. In order to make a clinical impact, the etoposide drivers will have to be validated in *ex vivo* models using AML patient samples, as well as in animal models.

Even though modulators generally exhibited synergy and mediators generally exhibited standalone cytotoxicity, their effect was not alike in all AML cell lines. None of the common AML oncogenic alterations correlated with observed response. However, few etoposide-evoked changes were in agreement with the response. For example, the absence of synergy between GDC-0152 (targeting the modulator BIRC5) and etoposide in MV-4-11 and OCI-AML3 cell lines may have resulted from bypassing TP53-mediated apoptosis because of high expression of *CDKN1A* (P21) and *MDM2* after etoposide treatment. Furthermore, high expression *BRCA1* in HL-60 cells may have contributed to efficient DNA double strand repair and absence of synergy between nicotinamide (targeting the modulator PARP9) and etoposide. Along the same line, presence of synergy between sabutoclax (targeting the mediator BCL2A1) and etoposide may have resulted from high expression of *BAX* after etoposide treatment in MOLM-13 cells. Finally, the highest basal expression of *PLK1* and etoposide-driven dynamics may have contributed to observed synergy between volasertib (targeting the mediator PLK1) and etoposide.

Similar studies could be conducted with other classical cancer drugs. New anti-cancer drugs provide limited clinical benefits and they are prohibitively costly. We envision fine-tuning and individualization of established chemotherapies based on their transcriptional response and resulting changes to cellular dynamics.

## MATERIALS AND METHODS

### Cell culture and drug treatment

Acute Myeloid Leukemia (AML) cell lines F-36P, HL-60, KASUMI-1, MOLM-13, MONO-MAC-6, MV-4-11, NB-4, NOMO-1, OCI-AML2, OCI-AML3, and THP-1 were purchased from Deutsche Sammlung von Mikroorganismen und Zellkulturen (DSMZ, Germany). Cell lines were maintained at 37°C and 5% CO_2_ in appropriate media (Supplementary Table 1). 293T cells were cultured in DMEM (Gibco, Germany) along with 10% FBS (Biochrom, Germany). Cell lines were routinely verified for mycoplasma contamination using Venor^®^GeM Mycoplasma Detection Kit (Sigma-Aldrich, Germany). Cell lines were authenticated by Multiplexion, Germany. The inhibitors were purchased from Abcam (UK), Biozol (Germany), and Santa Cruz Biotechnology (US).

### WST-8 cell viability assay

Reduction of WST-8 by cellular dehydrogenases produce formazan, whose signal is directly proportional to the number of viable cells. Both reduced cell proliferation and cell loss due to drug toxicity diminish the WST-8 signal. We seeded 1 × 10^4^ cells per well in a 96-well plate and incubated overnight. Cells were then treated for 24 hours with various concentrations of etoposide (0.02, 0.05, 0.1, 0.2, 0.37, 0.78, 1.56, 3.13, 6.25, 12.5, 25, 50 μM). Cell viability was measured using a colorimetric cell viability kit (WST-8) from PromoKine, Germany. In short, after the treatment with etoposide, 10% WST-8 reagent was added to the cells. After 1-4 hours incubation in dark at room temperature, absorbance was measured at 450 nm using Spectramax iD3 (Molecular Devices, USA) spectrometer. Absorbance from the DMSO-treated cells (vehicle control) was considered as 100% cell viability and used to calculate percentage cell viability after etoposide treatment.

### Annexin V apoptosis assay

We seeded 2 × 10^5^ cells/ml in a 6-well plate and incubated overnight. Cells were then treated with cell line-specific etoposide IC_50_ concentrations, derived from the cell viability assay, for 24 hours, washed twice with ice-cold PBS, and resuspended in binding buffer (1 × 10^6^ cells/ml). Thereafter, 100 μl of cell suspension (1 × 10^5^ cells) was transferred to a new tube, followed by addition of 5 μl each of Annexin V and PI staining solution (FITC Annexin V apoptosis detection kit I, BD Biosciences, USA). Cells were then gently vortexed and incubated in dark for 15 minutes at room temperature. 400 μl of binding buffer was then added to the cells and analyzed using BD Accuri C6 flow cytometer (BD Biosciences, USA).

### RNA-Seq: RNA extraction and library preparation

The gene expression profiles in 11 untreated and etoposide-treated AML cell lines were determined by RNA sequencing. 1 × 10^6^ cells per well were seeded in a 6 well plate containing 5 ml of the media. Cells were incubated overnight and then treated for 24 hours with etoposide at cell line-specific IC_50_ concentrations. Cells from 3 wells were then pooled together and total RNA was isolated using TriFast, peqGOLD total RNA kit and DNase I Digest kit (VWR PEQLAB GmbH, Germany) according to manufacturer’s instructions. The quality and integrity of the extracted RNA was examined using a 2100 Bioanalyzer (Agilent technologies). Samples were sequenced by Illumina HiSeq 2000 using TruSeq stranded mRNA HT sample prep kit at the Genomics Core Facility at the Institute of Molecular Biology (IMB, Mainz, Germany). The targeted sequencing depth was 30 million reads.

### RNA-Seq: analysis

We assessed the quality of raw sequencing reads using FastQC (Babraham Bioinformatics, Cambridge, UK). We then mapped these reads to the human reference genome (gencode release 25 GRCh38. p7) using the STAR aligner (v2.5.3a) [36], with the option “–quantMode GeneCounts” to count the number of reads mapped per gene. Quality of the expression data was assessed using NOISeq (v2.20.0) [37] R package [38]. We then performed differential gene expression analysis using edgeR (v3.20.1) [39]. Genes with fold change higher than 2 folds and FDR below 0.05 were considered as differentially expressed. Data is available at GEO Series accession number GSE126895 [40].

### Weighted gene co-expression network analysis (WGCNA)

To identify modulators and mediators of etoposide sensitivity, we performed weighted gene co-expression network analysis (WGCNA) using basal gene expression in AML cell lines prior and after etoposide treatment. Prior to WGCNA analysis, raw RNA-Seq counts were subjected to trimmed mean of M values (TMM) normalization, a preferred method for between-sample normalization [41]. The resulting co-regulated networks were compared to identify genes: (i) co-regulated only before treatment; (ii) co-regulated only after treatment; and (iii) unaffected by treatment. Gene Ontology analysis was performed for identified networks using the Database for Annotation, Visualization and Integrated Discovery (DAVID, https://david.ncifcrf.gov/). Cell line specific expression levels of co-regulated genes unaffected by treatment were correlated with cell specific etoposide IC_50_ concentrations by Pearson correlation statistics using the WGCNA package in R [12]. The co-regulated genes with positive and negative correlation with etoposide IC_50_ were selected for Gene Ontology analysis using DAVID.

### Identification of mediators among etoposide-evoked gene expression changes

The Project Achilles (PAch) [42] dataset was utilized to retrieve genes most likely to be essential for AML cell survival. PAch investigated the effect of more than 11k shRNA-mediated individual gene knockdowns on cell survival in 501 cancer cell lines, including all AML cell lines used in the present study. Genes with negative DEMETER scores (defined in a previous study [14]) were considered essential for cancer cell survival. Genes essential for 6 or more AML cell lines as well as differentially expressed after etoposide treatment were considered potential essential mediators and experimental validated.

### Prediction of etoposide emulators

Emulators, i.e., gene modulations and compounds that evoke gene expression changes similar to those evoked by etoposide, were identified using the Connectivity Map (CMap, Broad Institute) [43]. CMap provides changes in the expression of 1000 genes following gene perturbations and treatments with numerous small-molecule compounds. These genes and drugs were identified by uploading the top 300 overlapping etoposide-evoked gene expression changes (150 up- and 150 down-regulated) from AML cell lines to CMap via the CLUE platform (CMap and LINCS Unified Environment).

### Driver validation using inhibitors

The inhibitors against the selected drivers were identified using the GeneCards [44], IUPHAR/BPS guide to pharmacology [45], and CMap [43] resources. These drivers were then validated using WST-8 cell viability assay. AML cell lines were treated for 24 hours with 1 nM, 100 nM, and 10 μM of each inhibitor alone, as well as in combinations with cell-specific IC_25_ concentrations of etoposide, followed by WST-8 cell viability assay. Percentage cell viability compared to vehicle-treated cells, taken as 100%, was calculated for single and combination treatments. For combination treatment screening, the synergy was defined as per-response additivity approach [46]. The combination index (CI) was calculated as $CI=\frac{E_{A}+E_{B}}{E_{AB}}$ where E_A_ is the effect of inhibitor A, E_B_ is the effect of etoposide and E_AB_ is the effect of combination of inhibitor A and etoposide. CI < 1 was considered as synergy with etoposide, while CI > 1 was considered as antagonism, and CI = 1 was considered as additive effect.

### Driver validation using shRNA-mediated gene knockdown

To investigate the effect of individual gene knockdowns on AML cell survival, we cloned shRNA targeting *BCL2A1*, *IGFIR*, and *ROCK1* into Tet-pLKO.1-puro vector (kindly provided by Dimitri Wiederschain, Novartis Institutes for BioMedical Research, Cambridge, MA). shRNA sequences were obtained from the PAch resource and were synthesized by Sigma-Aldrich, along with RHS4743 expressing scrambled shRNA (Supplementary Table 2). These sequences have been validated at the protein levels in several previous publications [47–49]. Lentiviral particles were generated by co-transfecting psPAX2, pMD2. G along with previously generated shRNA expressing vectors into 293T cells. Transfection was carried out using TransIT (Mirus) as per the manufacturer’s instructions. To achieve stable transduction, AML cell lines were seeded 1 × 10^6^ in a 6-well plate, with indicated virus supernatant in presence of 5 μg/mL polybrene and spin-infected at 2500 rpm at 32° C for 1 and 45 hours. Following 16 hours incubation at 37° C, cells were supplemented with 1–2 μg/mL puromycin (Sigma-Aldrich, Germany). Furthermore, to induce knockdown of the indicated drivers, we seeded 5 × 10^5^ cells per well in 6-well cell culture plates. We then induced the knockdown by treating the cells with doxycycline (200 ng/ml) and measured the cell viability after 24, 48, and 72 hours using the WST-8 assay. The effect of shRNA-mediated gene knockdown on cell viability was calculated by comparing doxycycline-untreated and -treated cells.

### DNA damage measurement using flow cytometry

To compare the amount of DNA damage caused by etoposide alone and in combination with other drugs, we measured the levels of phosphorylated H2A.X in HL-60 cells using flow cytometry. We stained the fixed HL-60 cells using the H2A.X phosphorylation assay kit (Merck, Germany) according to manufacturer’s instructions. In short, 5 × 10^5^ HL-60 cells were seeded per well in a 6-well plate and incubated overnight. Cells were treated for 24 hours with IC_25_ concentration of etoposide alone and in combination with other drugs. Next, cells were harvested and washed with PBS followed by fixation. Cells were then stained with either FITC-conjugated anti-phospho-Histone H2A.X (Ser139) or with the negative control mouse IgG-FITC conjugate for 20 minutes on ice. The amount of H2A.X was then measured using BD Accuri flow cytometer. The data was then analyzed using FlowJo software (v10).

### TCGA survival analysis

We retrieved the raw gene expression counts for 151 AML patients from The Cancer Genome Atlas (TCGA) through the Broad GDAC Firehose, along with the clinical data, using the R package RTCGAToolbox (v2.8.0) [50]. We then performed univariate survival analysis comparing the groups with high expression (above median) and low expression (below median) of selected drivers. We generated Kaplan-Meier plots with p-values calculated using Log-rank test. The comparison between gene expression in AML patients, from TCGA, and normal blood samples, from The Genotype-Tissue Expression (GTEx) [51], was performed using Gene Expression Profiling Interactive Analysis (GEPIA) web server [52].

### Statistical analysis

Unless otherwise specified, the experiments reflect 3 biological replicates. Data was analyzed using R language packages and GraphPad Prism software (v7). Graphs were plotted as mean ± SD. The etoposide IC_50_ concentrations were calculated using GraphPad Prism software by fitting the dose response curve by non-linear regression. Shapiro-Wilk test was performed to determine normal distribution for parametric tests. Two-way ANOVA with Benjamini and Hochberg FDR correction was performed to identify inhibitors with significant cytotoxicity [53]. Multiple t-tests with Benjamini and Hochberg FDR correction were performed to identify significant gene expression change between resistant and sensitive AML cell lines. One-way ANOVA with Dunnett’s multiple comparisons test was performed to identify significant γH2AX formation and cell death induction with drug-combinations in comparison to etoposide alone. Two-way ANOVA with Dunnett's multiple comparisons test was performed to identify statistically significant cytotoxicity after shRNA-mediated gene knockdown in comparison to cells treated with scrambled shRNA.

### Availability of data and material

The RNA sequencing data included in this work have been deposited in NCBI’s Gene Expression Omnibus (GEO) database with the accession number GSE126895 (https://www.ncbi.nlm.nih.gov/geo/) [40].

## SUPPLEMENTARY MATERIALS





## Abbreviations

AML: acute myeloid leukemia
CI: combination index
CMap: connectivity map
DAVID: database for annotation, visualization and integrated discovery
DMSO: dimethyl sulfoxide
FDR: false discovery rate
FITC: fluorescein isothiocyanate
GEPIA: gene expression profiling interactive analysis
GTEx: The Genotype-Tissue Expression
PAch: project achilles
PBS: phosphate-buffered saline
PI: propidium iodide
shRNA: short hairpin RNA
TCGA: The Cancer Genome Atlas
TOP2: topoisomerase II
WGCNA: weighted gene co-expression network analysis
