# Supplementary material for: Drivers of topoisomerase II poisoning mimic and complement cytotoxicity in AML cells
Source: Oncotarget. 2019 Sep 3;10(51):5298–312. doi: 10.18632/oncotarget.27112 (PMC6731103; doi:10.18632/oncotarget.27112)
Supplement: Supplementary file 1 [file oncotarget-10-5298-s001.pdf]

Drivers of topoisomerase II poisoning mimic and complement cytotoxicity in AML cells

SUPPLEMENTARY MATERIALS

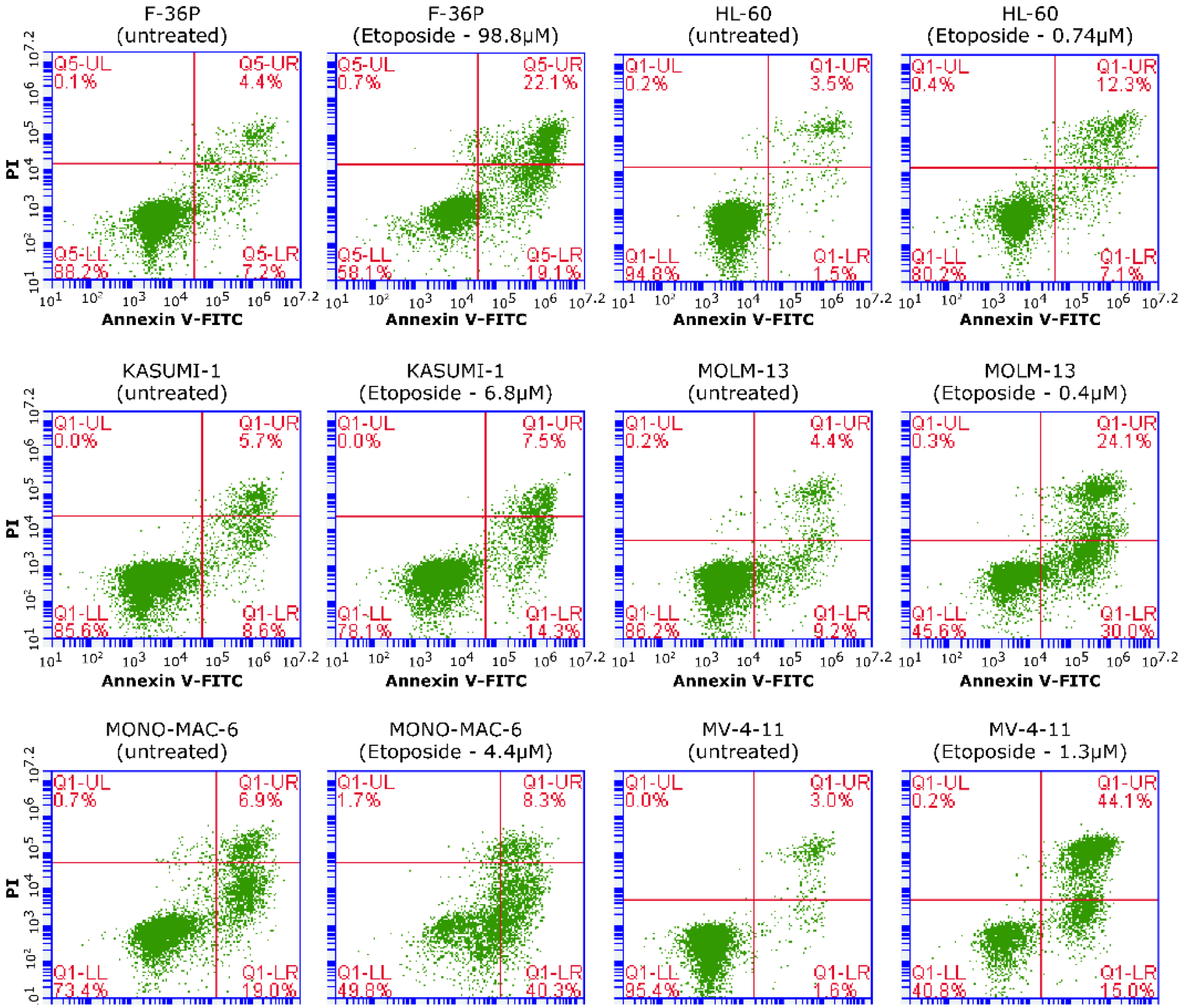

Supplementary Figure 1: Percentages of apoptotic AML cells in response to etoposide treatment. All AML cell lines were treated with cell line-specific IC_{50} concentrations of etoposide for 24 hours, followed by Annexin-FITC and PI staining, and detection by flow cytometry. Quadrant LL represents healthy, LR early apoptotic, and UR late apoptotic cells.

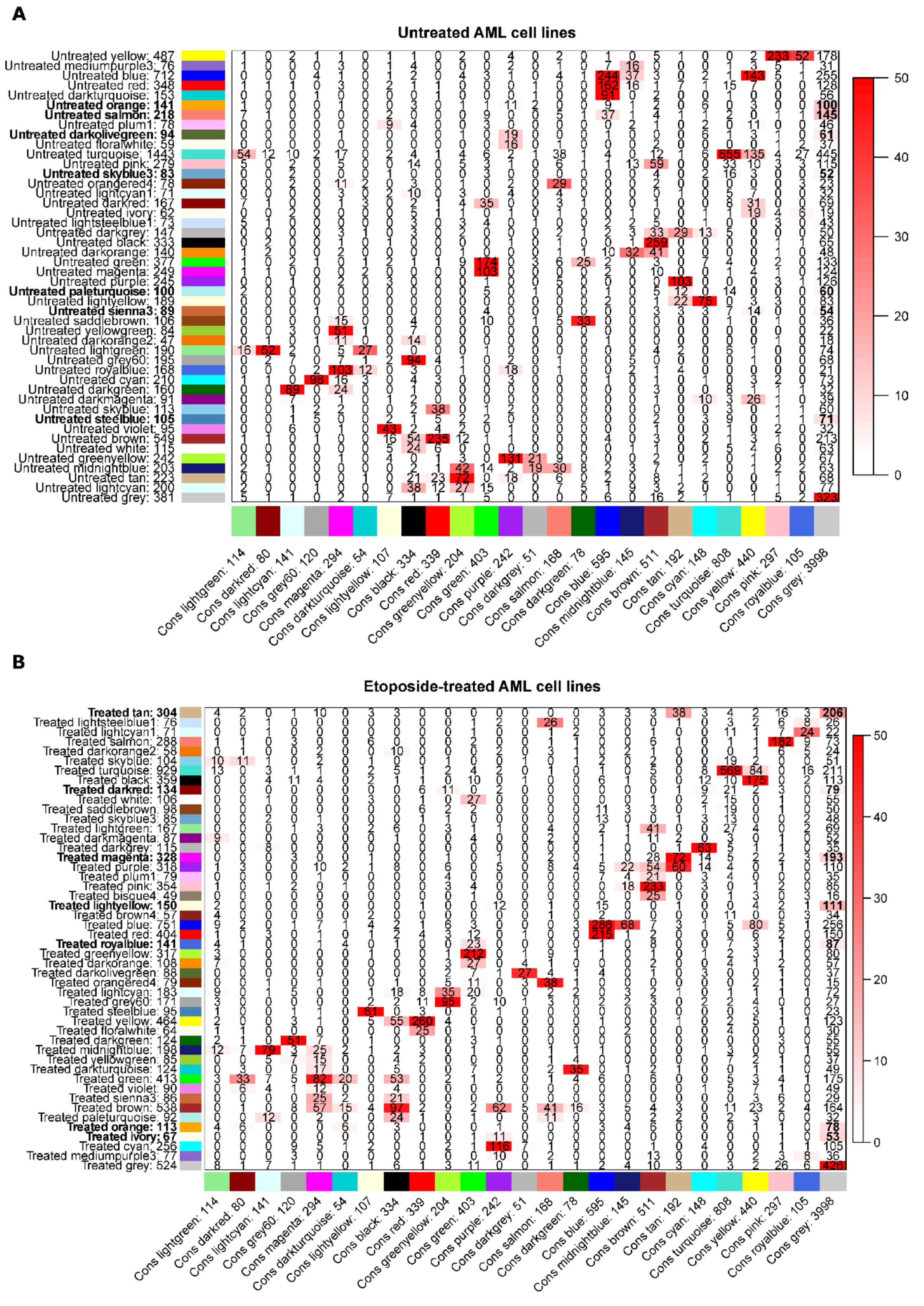

Supplementary Figure 2: WGCNA consensus network analysis. (**A**) Comparison of co-expression modules identified solely in untreated AML cell lines with consensus modules (present in both untreated and etoposide-treated AML cell lines). (**B**) Comparison of co-expression modules identified solely in etoposide-treated AML cell lines with consensus modules (present in both untreated and etoposide-treated AML cell lines). Color names on X- and Y-axis represent individual co-expressing modules and the number next to the module represent total number of co-expressing genes identified in that particular module. Numbers in the heatmap represent number of genes common to consensus network on the X-axis, while the number of genes to extreme right (common to Cons grey module) depicts the co-expressing genes unique to untreated AML cells.

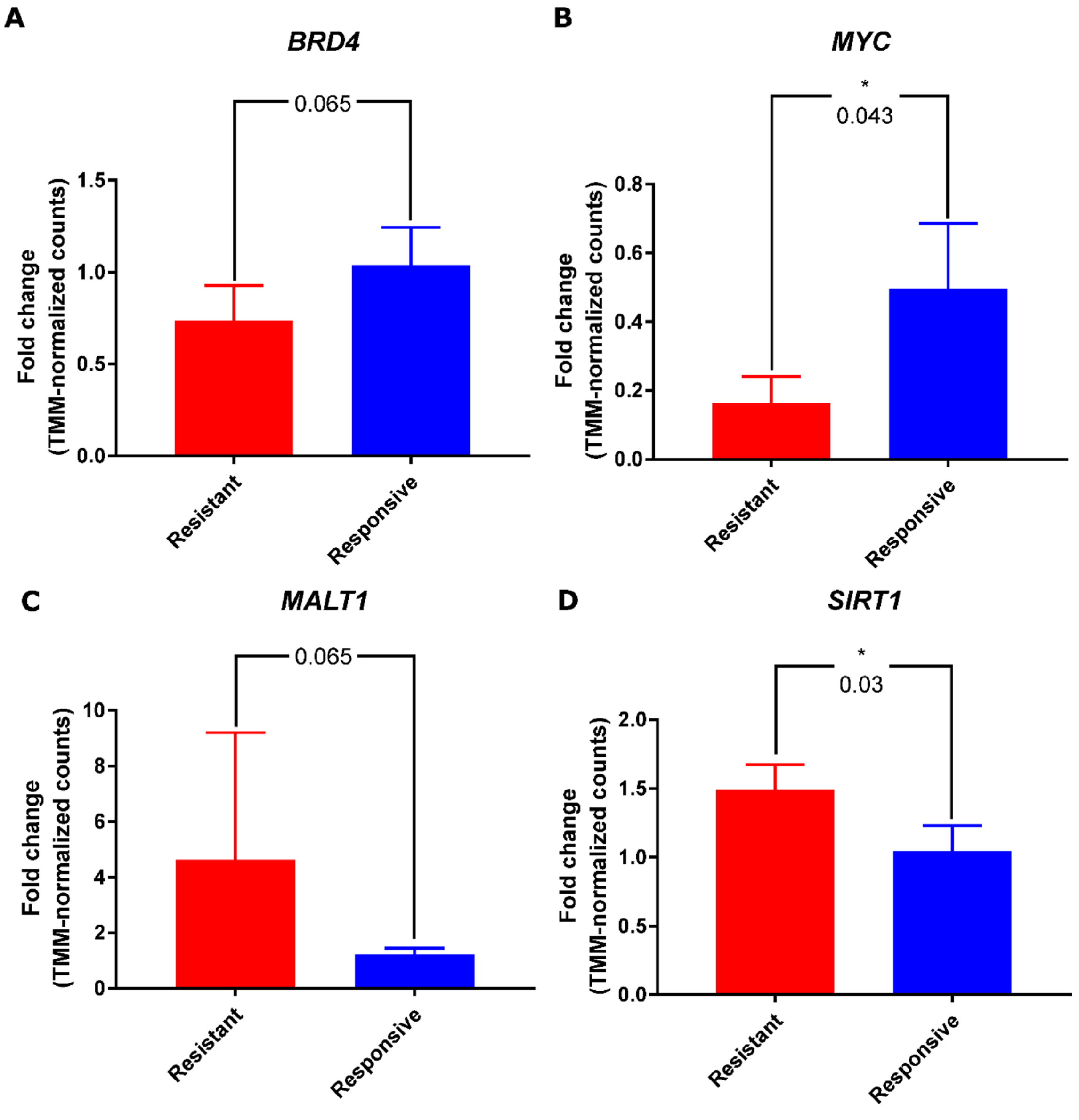

Supplementary Figure 3: Etoposide-evoked changes in levels of co-expressing genes. Cell lines exhibiting IC_{50} concentrations above third quartile (75%) (F-36P, KASUMI-1, and MONO-MAC-6) were considered resistant, while remaining AML cell lines were considered etoposide-responsive. (**A**) *BRD4* and (**B**) *MYC* repression after etoposide treatment in resistant cell lines. (**C**) *MALT1* and (**D**) *SIRT1* induction after etoposide treatment in resistant cell lines. Mann-Whitney test was performed to identify significant expression change between resistant and sensitive AML cell lines. Multiple t-tests with Benjamini and Hochberg FDR correction were performed to identify significant gene expression change between resistant and sensitive AML cell lines (indicated by asterisks, *FDR < 0.05).

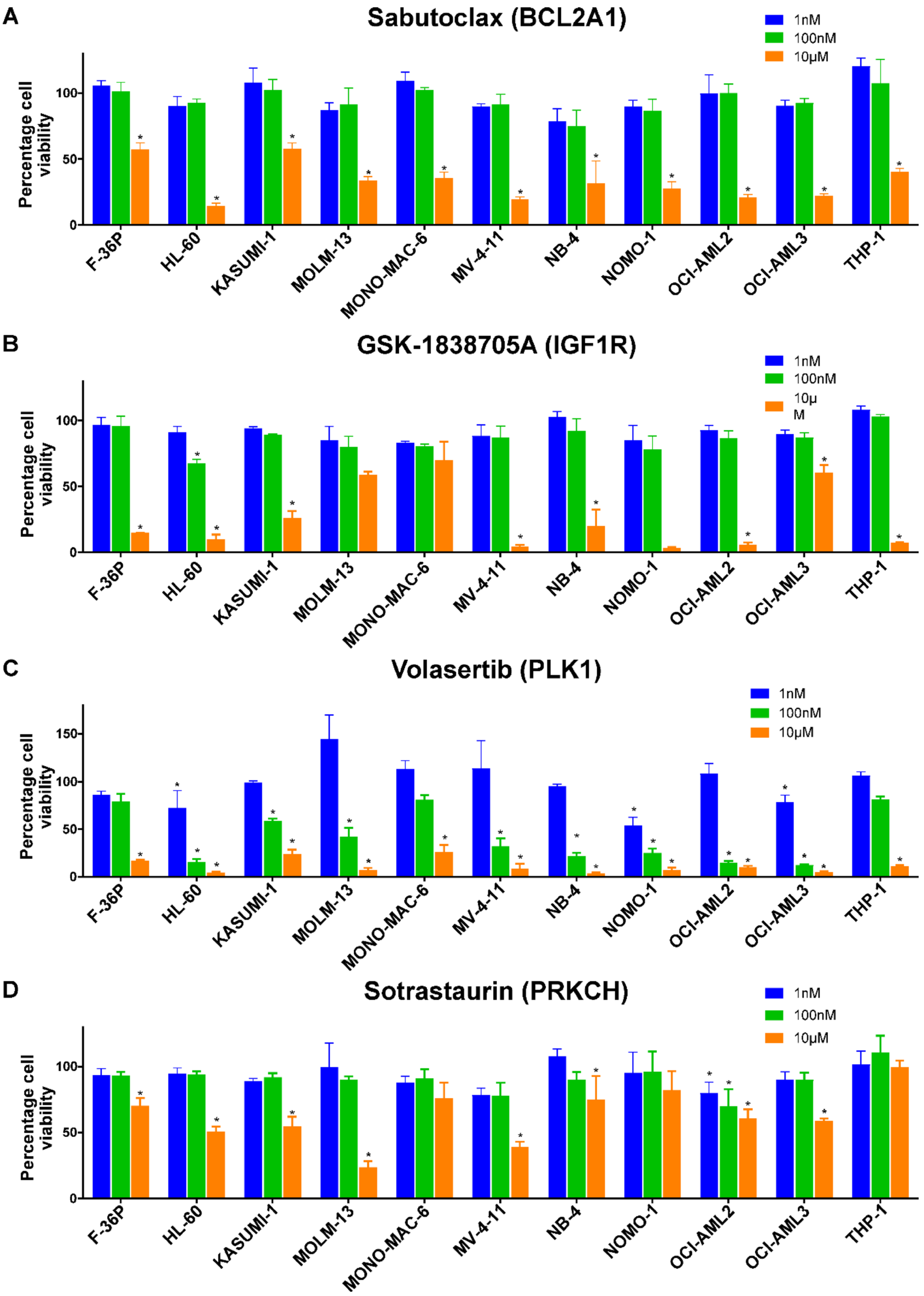

Supplementary Figure 4: Viability of AML cell lines in response to inhibitors targeting the essential mediators. (**A**) sabutoclax (BCL2 inhibitor), (**B**) GSK-1838705A (IGF1R inhibitor), (**C**) volasertib (PLK1 inhibitor), and (**D**) sotrastaurin (PRKCH inhibitor). Two-way ANOVA with Benjamini and Hochberg FDR correction was performed to identify statistically significant cytotoxicity in comparison to vehicle treated cells (indicated by asterisks, *Adj. $P < 0.05$). Data are represented as mean ± SD. Data are represented as mean ± SD.

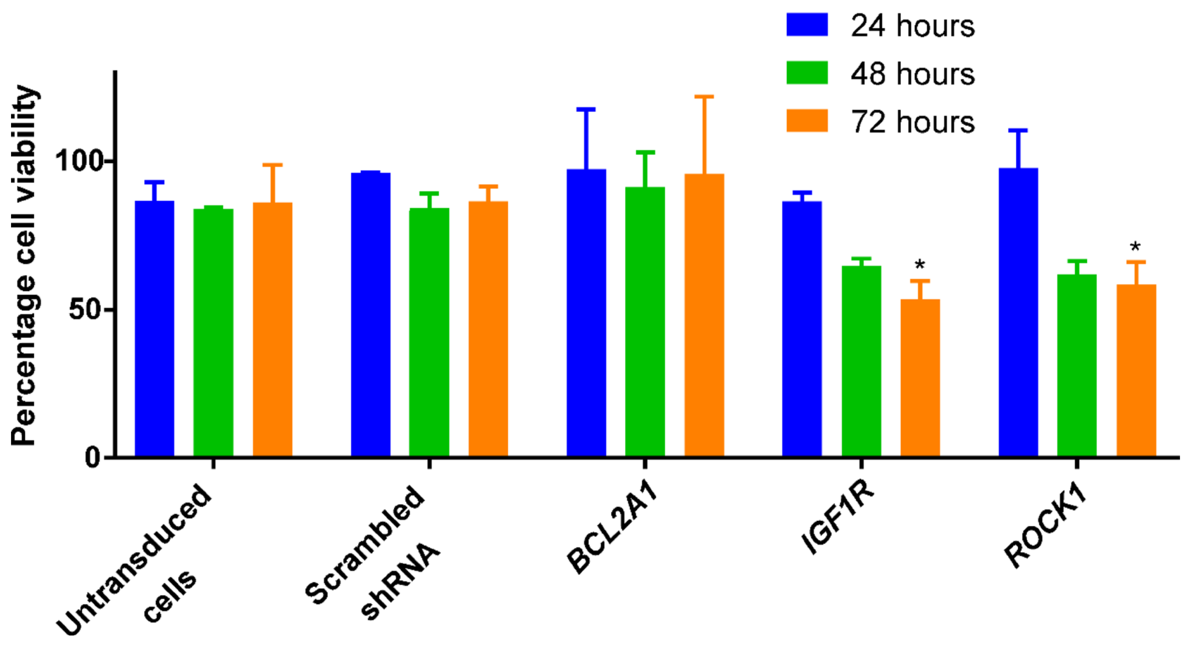

Supplementary Figure 5: Viability of HL-60 cells after shRNA-mediated gene knockdown of etoposide drivers *BCL2A1*, *IGF1R*, and *ROCK1* for 24, 48, and 72 hours. Two-way ANOVA with Dunnett's multiple comparisons test was performed to identify statistically significant cytotoxicity in comparison to cells treated with scrambled shRNA (indicated by asterisks, *Adj. $P < 0.05$). Data are represented as mean ± SD. Data are represented as mean ± SD.

Supplementary Table 1: Overview of the AML cell lines used in the study with their culture conditions

Cell lines	AML classification	Growth medium	Fetal calf serum (heat inactivated)	Supplements	Growth condition	Etoposide IC_{50} (µM)	Etoposide IC_{25} (µM)
F-36P	AML M6	RPMI 1640	20%	10 ng/ml granulocyte-macrophhage colony stimulating factor	37° C, 5% CO_2	98.81	25.96
HL-60	AML M3		10%	-		0.74	0.48
KASUMI-1	AML M2		20%	-		6.80	1.43
MOLM-13	AML M5		10%	-		0.39	0.12
MONO-MAC-6	AML M5		10%	MEM Non-essential amino-acid solution, 100 mM Na-pyruvate, 10 µg/ml human insulin		4.39	2.34
MV-4-11	AML M5		10%	-		1.33	0.36
NB-4	AML M3		10%	-		0.50	0.25
NOMO-1	AML M5		10%	-		1.65	0.95
OCI-AML2	AML M4	alpha-MEM	20%	-		0.29	0.16
OCI-AML3	AML M4		20%	-		1.00	0.58
THP-1	AML M5	RPMI 1640	10%	-		1.01	0.63

Etoposide IC_{50} and IC_{25} concentrations, after 24 hours treatment, were derived from WST8-based cell viability using GraphPad Prism software by fitting the dose response curve by non-linear regression.

Supplementary Table 2: Targets investigated using shRNA-mediated gene knockdown

Target gene	Clone ID (GPP)	Target sequence
BCL2A1	TRCN0000033496	GTTGCGGAGTTCATAATGAAT
IGF1R	TRCN0000039675	GCCGAAGATTTCACAGTCAAA
ROCK1	TRCN0000002161	CAGTACCAAATAGAGGAAATA

shRNA sequences were obtained from Project Achilles and Gene Perturbation Platform (GPP) resources.

Supplementary Table 3: Biological processes corresponding to the genes whose co-regulation was unaffected by the etoposide treatment in AML cell lines. See Supplementary Table 3

Supplementary Table 4: Table representing synergy with etoposide of the drugs inhibiting selected drivers in AML cell lines

**Drug (driver)**	**Treatment concentration (µM)**	**Combination index (CI)**										
		F-36P	HL-60	KASUMI-1	MOLM-13	MONO-MAC-6	MV-4-11	NB-4	NOMO-1	OCI-AML2	OCI-AML3	THP-1
GDC-0152 (BIRC5)	10	0.7	0.9	1.1	0.9	1	1.4	0.9	1.4	1.3	1.3	1.2
	0.1	0.7	0.9	1.1	0.8	0.8	1.2	1	1	1.1	1.3	0.8
	0.001	0.8	0.7	1	0.7	0.8	1.2	1	0.8	1	1.2	0.8
GSK-1838705A (IGF1R)	10	1.4	1	1.2	1.4	1.3	1.6	0.5	1.3	1.3	1.5	0.8
	0.1	1.2	1.4	1.1	1.2	1.4	1.3	0.9	1.2	1.4	1.4	0.7
	0.001	1.3	1.5	1.5	1.1	1.3	1.4	1.2	1.3	1.4	1.6	1.2
LY-3039478 (NOTCH1)	10	3.2	1.2	1.3	0.7	1.3	1.3	1	1.3	1.1	1.6	1.3
	0.1	1.3	1.2	1.2	0.8	1.1	1.2	1.8	1.3	1.1	1.5	1.3
	0.001	10.3	1.3	1.3	0.8	1.1	1.5	1.2	1.3	1.1	1.7	0.8
Nicotinamide (PARP9)	10	1	1	1.2	0.9	1.5	1.9	1.2	2.3	1.3	1.2	1
	0.1	0.8	1.1	1.2	0.8	1.1	0.6	0.7	1.4	0.9	1	0.9
	0.001	0.6	1.1	1	0.8	1.3	0.6	0.7	0.9	0.9	1	0.8
Rapamycin (mTOR)	10	1.8	1.3	1.6	0.9	1.4	0.5	1.2	1.4	1.5	1.3	1.1
	0.1	1.1	1.6	1.6	0.9	1.1	1	2.3	1.1	1.5	1.2	0.9
	0.001	1.9	1.4	1.4	1.1	1	0.8	1	0.9	1.1	1.3	1.1
Rockout (ROCK1)	10	1	0.9	1.2	0.7	0.8	0.7	1.2	0.8	1.4	1.4	1.1
	0.1	1.2	0.7	1.3	1.1	0.9	0.5	1.3	0.7	1.4	1.6	1
	0.001	1.1	1	1.3	0.6	1	0.7	1.5	1.1	1.3	1.5	1.3
Sabutoclax (BCL2A1)	10	-	1.1	1.8	0.8	1.2	1.4	1.5	1.4	1.1	1.6	1.7
	0.1	-	1.3	1.5	0.9	1.1	1.3	1.5	1.7	1.3	1.4	1.6
	0.001	1.2	1.5	1.6	1.3	1.5	1.4	1.3	1.4	1.6	1.6	1.3
Sotrastaurin (PRKCH)	10	1.4	1.1	1.2	0.9	1.3	1.4	1.5	1.4	1.4	1.3	0.8
	0.1	1.1	1	1.2	0.7	1	1.5	1.1	1.1	1.5	1.3	0.8
	0.001	1.3	1.5	1.5	1.2	1.2	1.3	1.2	0.8	1.2	1.6	0.6
TWS-119 (MYC)	10	1.6	0.9	1.4	1.1	1.4	1.2	1	1.1	1.4	1.4	0.8
	0.1	4.3	1.3	1.5	1.2	1.4	1.2	1.7	1.4	1.6	1.3	1.1
	0.001	4.4	2.8	1.6	1.3	1.4	1.5	1.9	3.6	1.6	2.1	1.4
Volasertib (PLK1)	10	20.1	1.3	1.1	1.2	1.4	2.6	0.5	3	1.7	1.5	1.2
	0.1	2.8	2.3	2	2	2	15.2	2	5.2	1.8	3	1.4
	0.001	1.3	1.4	1.6	1.2	1.4	1.2	1.2	1.3	1.3	1.4	1.4
Vorinostat (HDAC)	10	1.3	1.1	1.1	0.7	1.7	0.6	0.7	1.1	0.9	0.9	0.9
	0.1	0.9	1.1	1.1	0.6	1.1	0.5	0.5	0.7	1.1	0.8	0.7
	0.001	1.1	1.4	1.5	1.1	0.6	1.2	1.1	0.7	1.2	1.2	0.8

Cells were treated with 3 concentrations of each drug in combination with IC25 concentration of etoposide for 24 hours. Combination index (CI) was calculated to determine either synergistic (CI<1), additive (CI=1), or antagonist (CI>1) effects.

Supplementary Table 5: Table representing standalone cytotoxicity of the drugs inhibiting selected drivers in AML cell lines. See Supplementary Table 5

Supplementary Table 6: Biological processes corresponding to the genes co-regulated only in untreated AML cell lines. See Supplementary Table 6

Supplementary Table 7: Biological processes corresponding to the genes co-regulated only in etoposide-treated AML cell lines. See Supplementary Table 7

Supplementary Table 8: Etoposide-evoked gene expression changes in AML cell lines

Cell lines	Numbers of gene expression changes	Numbers of induced genes (%)	Numbers of repressed genes (%)
F36-P	4615	3882 (84.1)	733 (15.9)
HL-60	1007	875 (86.9)	132 (13.1)
KASUMI-1	3558	2874 (80.8)	684 (19.2)
MOLM-13	1643	1284 (78.2)	359 (21.9)
MONO-MAC-6	1788	1307 (73.1)	481 (26.9)
MV-4-11	2091	1883 (90.1)	208 (10.0)
NB-4	2383	1965 (82.5)	418 (17.5)
NOMO-1	1679	1177 (70.1)	502 (29.9)
OCI-AML3	1215	1028 (84.6)	187 (15.4)
THP-1	1278	1050 (82.2)	228 (17.8)
	Average	81.30%	18.80%

The changes were identified by differential expression analysis using edgeR comparing the untreated AML cell lines with etoposide-treated at cell -specific IC_{50} concentrations.

Supplementary Table 9: Essential effectors in AML cell lines

Cell lines	Numbers of etoposide-evoked gene expression changes	Number of predicted essential genes (%)	Repressed predicted essential genes (%)	Induced predicted essential genes (%)
F-36P	4615	1447 (31.3)	239 (16.5)	1208 (83.5)
HL-60	1007	348 (34.6)	45 (12.9)	303 (87.1)
KASUMI-1	3558	1234 (34.7)	227 (18.4)	1007 (81.6)
MOLM-13	1643	549 (33.4)	118 (21.5)	431 (78.5)
MONO-MAC-6	1788	602 (33.7)	144 (23.9)	458 (76.1)
MV-4-11	2091	702 (33.6)	63 (9)	639 (91)
NB-4	2383	762 (32)	128 (16.8)	634 (83.2)
NOMO-1	1679	580 (34.5)	156 (26.9)	424 (73.1)
OCI-AML3	1215	416 (34.2)	55 (13.2)	361 (86.8)
THP-1	1278	417 (32.6)	71 (17)	346 (83)

The numbers and percentages of etoposide-evoked gene expression changes essential for AML cell survival identified using Project Achilles resource.

Supplementary Table 10: Mediators predicted to contribute to etoposide-mediated cytotoxicity. See Supplementary Table 10

Supplementary Table 11: Emulators evoking gene expression changes either similar (etoposide-like) or opposite (etoposide-contrary) to those evoked by etoposide. See Supplementary Table 11
